# A human sentinel surveillance framework for comprehensive exposure assessment in occupational and environmental health

**DOI:** 10.3389/fpubh.2025.1641884

**Published:** 2025-10-08

**Authors:** Emine Aktas, Kaoutar Chbihi, Hilde De Raeve, Janne Goossens, Lode Godderis

**Affiliations:** 1 Independent Researcher, Istanbul, Türkiye; 2 Environment and Health Unit, Department of Public Health and Primary Care, Faculty of Medicine Katholieke Universiteit Leuven, Leuven, Belgium; 3 Human Epidemiology and Environmental Health Team, Faculty of Sciences, Moulay Ismail University, Meknes, Morocco; 4 IDEWE, External Service for Prevention and Protection at Work, Heverlee, Belgium

**Keywords:** environmental exposure, occupational exposure, exposure assessment, human biomonitoring, exposome, sentinel surveillance, risk assessment, data analysis

## Abstract

Environmental and occupational exposures are increasingly recognized as major determinants of population health, contributing to the rising burden of chronic diseases and adverse health outcomes, yet traditional surveillance systems are often inadequate for capturing the complex and evolving nature of human exposures across diverse settings. In response, we propose the Human Sentinel Surveillance Platform (HSSP), a new digital infrastructure based on sentinel surveillance framework, to monitor exposures and health effects in real-time and via trained and motivated health professionals in order to identify emerging exposure trends. This perspective paper defines the foundational pillars, data governance principles, and operational workflows of the HSSP, while critically examining its potential impact on health policy, practice, and exposome research. The platform integrates biomarker-based monitoring, validated questionnaires, and adaptive protocols that can be updated in response to new threats, ensuring methodological relevance over time. Its four foundational pillars include: (1) a structured network of health care professionals, (2) targeted training and capacity building, (3) harmonized data collection using standardized tools, and (4) secure data repository and management aligned with ethical and regulatory standards. By incorporating multidisciplinary data from epidemiology, toxicology, genetics, and exposure science, HSSP enables comprehensive exposure characterization, longitudinal analysis of exposure-health relationships, early warning and timely public health regulatory and preventive interventions. This scalable and adaptable platform bridges critical data gaps in exposome research by capturing dynamic human-environment interactions and generating actionable insights to inform targeted interventions and provide evidence-based foundations for public health policy.

## 1 Introduction

Environmental factors and stressors, namely air pollutants, noise, artificial light, heat and climate change, are being increasingly associated with several non-communicable diseases (1). Additionally, occupational exposure to hazardous chemicals such as benzene, asbestos, and silica contributes to over 1.9 million work-related deaths annually worldwide (2), underscoring the pervasive and multifaceted nature of environmental and occupational exposure. Environmental or occupational health-related risks are predominantly investigated through reactive research studies or narrowly targeted surveys, which limits risk assessment to isolated health outcomes, specific environmental factors, or fragmented population groups. This approach fails to capture the dynamic interplay of exposures across the exposome, perpetuating systemic gaps in real-time data and hindering comprehensive risk mitigation strategies.

Current occupational and environmental surveillance approaches face significant challenges that human sentinel surveillance systems uniquely address. Traditional monitoring methods often suffer from delayed detection of emerging hazards, relying heavily on retrospective data analysis that fails to capture the real-time dynamic variability of exposures across settings and populations. In contrast, sentinel-based approaches generate continuous and adaptive data streams that integrate exposure measurements with health outcomes, allowing fluctuations and trends to be monitored as they occur and enabling earlier identification of evolving risks and timely preventive action. Moreover, existing approaches frequently operate in silos-separating occupational from environmental monitoring and disconnecting exposure assessment from health outcome tracking-which prevents a comprehensive understanding of exposure-disease relationships (3, 4). These conventional systems typically focus on known contaminants with predefined thresholds, overlooking novel compounds, complex chemical mixtures, and rapidly emerging risks (5, 6). Resource constraints limit the geographic and demographic scope of surveillance, creating blind spots in vulnerable or underserved populations (7).

In contrast, Human Sentinel Surveillance systems overcome these limitations by integrating real-time biomonitoring with health outcome tracking in strategically selected populations, enabling early detection of both known and emerging hazards. The Human Sentinel Surveillance Platform (HSSP) was specifically developed to address the known limitations of current occupational and environmental surveillance systems. Through this comprehensive and flexible framework, evidence-based interventions are supported and disciplinary barriers are bridged, complex exposure scenarios are captured, biomarkers and protocols can be updated, and timely evidence is provided for interventions before widespread health impacts occur, enabling the integration of interdisciplinary data, thereby creating an adaptive, responsive framework to protect public health in rapidly changing environmental and occupational context (7, 8). It overcomes limited geographic coverage by focusing on strategically selected sentinel populations and uses adaptive protocols to track novel or evolving hazards. Through the combination of biomonitoring, standardized questionnaires, and interdisciplinary data integration, HSSP provides a comprehensive solution that directly responds to the systemic weaknesses identified in current surveillance practices. Sentinel systems are known for potentially improving disease surveillance through monitoring and by informing on disease incidence the concept of sentinel surveillance refers to monitoring in which data is collected systematically from selected sites or populations to serve as early-warning indicators of health trends at large (8). Such sentinel surveillance approach can help in the early signaling of hazards and in the pre-identification of health problems at specific sites (9). In sentinel surveillance, data is collected from selected reporting sources that represent all cases of a defined condition (10), showing robustness for detecting specific public health issues and for prompt monitoring and investigation using limited resources (11). Sentinel surveillance systems have been successfully applied in the monitoring of chronic diseases such as cancer, diabetes, obesity, and mental health disorders within communities (12, 13). Additionally, the systems have been instrumental in guiding public health response during COVID-19 pandemic (14) and in tracking global infectious diseases, like influenza, human immunodeficiency virus (HIV), and hepatitis, helping health authorities to gauge disease prevalence and respond to health threats with data-driven interventions (15). Building on this established capacity in disease surveillance, the sentinel approach also provides a robust foundation for exposure assessment by enabling the systematic collection of high-quality, population-based data on environmental and occupational hazards. Reliable and high quality exposure data are required to understand the extent and the diversity of environmental and occupational exposure within communities. Applying sentinel surveillance approach in environmental and occupational health context will provide early warning signs of potential human exposure, that will also enhance systemic analysis and improve estimates about the health related outcomes (16). This has been shown through the Hazardous Chemical Products Register for Occupational use in Belgium (PROBE) study, which demonstrated the effectiveness of sentinel surveillance in collecting data on occupational exposures to hazardous chemicals such as benzene, toluene, diesel exhaust, and wood dust (16). Similarly, the French national cross-sectional survey of occupational risks ‘Surveillance Medicale des Risques’ (SUMER) study, has provided valuable epidemiological data on exposures to carcinogenic, mutagenic, and reprotoxic chemicals such as diesel engine exhaust, wood dust, and crystalline silica, using the same approach (17, 18).

In the line with these, the aim of this paper is to present the Human Sentinel Surveillance Platform (HSSP) as an innovative and strategic approach to strengthening occupational and environmental health surveillance system. It aims to establish a robust sentinel network of occupational health professionals to systematically collect and analyze reliable, real-time data on human exposures, enabling early signalization of emerging hazards and risks. By developing a standardized exposure database and identifying risky work environment and populations, HSSP serves as a critical tool for timely exposure and risk detection, informed decision-making, and targeted public health interventions. The platform and this paper further aims to characterize emerging occupational and environmental hazards across diverse work settings, develop a standardized exposure database to support longitudinal research, and enable timely detection and mitigation of health risks.

## 2 Materials and methods

### 2.1 Human Sentinel Surveillance Platform (HSSP) for exposure data collection

**Figure 1:**
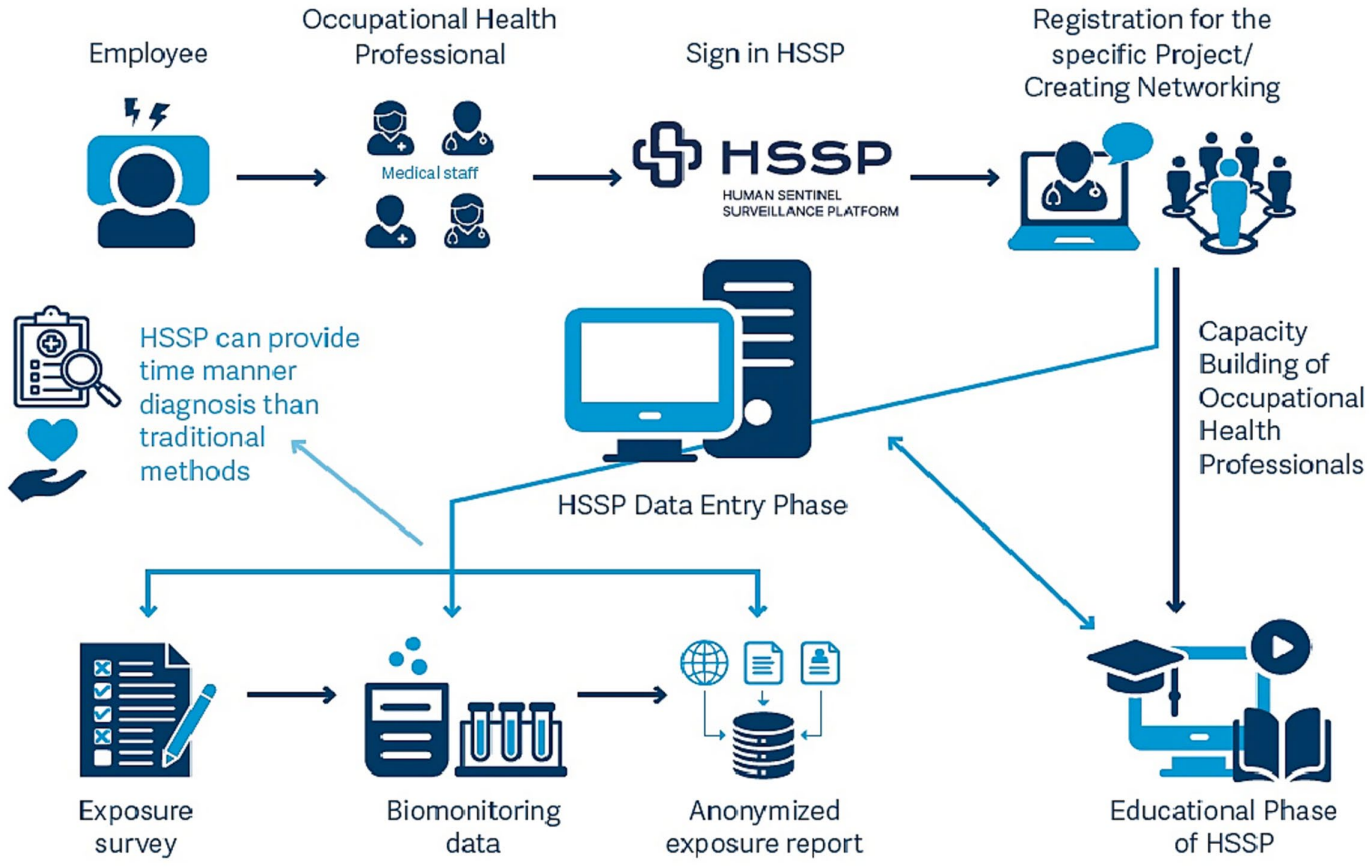
General framework for the applicability of the HSSP in occupational health assessment (Human sentinel surveillance platform).

The concept behind the development of the Human Sentinel Surveillance Platform (HSSP) is based on sentinel surveillance approach to address the growing need for reliable and standardized occupational and environmental health data, overcoming the limitations of existing systems that often lack timely and comprehensive monitoring of diverse workplace exposures and health risks. Therefore, we suggest a newly developed platform that aims to systematically collect data on occupational and environmental pollutants and high risk concern substances. The HSSP has the mission to gain valuable insights into the exposome, by gathering and analyzing exposure data. Human sentinel surveillance platform is based on a structured approach, designed to efficiently assess occupational and/or environmental exposures and their impacts in various settings. The steps, presented in Figure 1 and focusing on occupational health as an example, can be outlined as follows:

#### 2.1.1 Registration and network creation

As sentinel reporters occupational health and safety (OHS) professionals are the network of the platform and they play crucial role for collection of reliable and quality exposure data. Firstly, they and sign into the platform and apply to the project of interest in the HSSP. Upon application, they are requested to fill out a participation form that inform about their affiliation, working institution and sector as well as on work experience. The platform establishes a structured network of OHS professionals.

#### 2.1.2 Training and capacity building

Upon participation approval in HSSP, OHS professionals are invited to follow an online training modules based on the specific project, either through short videos or through elaborated training programs including standard operational procedures (SOP) for biomonitoring data and navigating usage of the platform. Training is required for all OHS professionals before data collection begins. This training promotes capacity building and reinforcement of skills among OHS professionals in environmental and occupational health, exposure assessment, sentinel surveillance, as well as sample and data collection. The training also helps guiding OHS professionals on the platform usage and data collection process.

#### 2.1.3 Data collection and entry

In the HSSP, Research Electronic Data Capture (REDCap) is used as the tool of reference to collect data, which is a software that allows building and managing online surveys and databases for research studies and projects, through a secure web application (19, 20). In this section, OHS professionals are directed to the survey of the project of interest and can collect data from the target population, with the respect of ethical principles and guidelines. The HSSP can also gather ethical documents (e.g., letter of information and informed consent) to approve prior to participation. Along with survey data, Human Biomonitoring data including matrices can also be collected with the respect of ethical guidelines and data anonymization process, depending on the projects’ objectives.

#### 2.1.4 Data management procedures and practical features of the HSSP

The HSSP is meticulously designed to ensure high standards of data integrity, privacy, and regulatory compliance. This comprehensive approach encompasses various stages of data handling as follows:

##### 2.1.4.1 Data privacy and confidentiality

Data can be collected on secure software hosting the survey of interest. Projects on the HSSP are accepted to use the platform if they are complying with the General Data Protection Regulation (GDPR) and if they are aligned with ethical principles, namely by using the informed consent as a form of participation approval by participants. Project-based ethical confirmation is obtained from relevant institutional review boards (IRBs) or ethical committees before data collection begins. On another hand, data collected through the HSSP are either anonymized or pseudonymized. This consists of replacing identifiers with unique codes, which helps protecting participant privacy, namely, by reducing re-identification risks and by supporting secure data sharing while allowing useful data analysis.

##### 2.1.4.2 Data storage and integrity

Data is securely managed and stored in the HSSP and in survey management software, namely REDCap, that employs robust security measures, including role-based access controls, data encryption, audit logs, and automatic backups, to protect sensitive information. In REDCap, confidential data are easily handled due to customizable permissions, user authentication options including two-factor authentication, and ability to de-identify data for privacy protection. Additionally, its self-hosted option allows institutions to maintain direct control over data storage and security policies.

##### 2.1.4.3 Human biological monitoring data handling

Registered OHS professionals enter sample IDs and lab names on REDCap without linking them to personal identifiers, maintaining strict privacy and anonymity. The source data is securely stored in compliance with GDPR and national laws. All staff members handling data are requested to sign confidentiality agreements. Moreover, trial documents are archived in line with ICH-GCP E6 (R2) guidelines, ensuring data integrity and accessibility for 25 years post-termination.

Biomonitoring data are processed and analyzed accredited central laboratories that have quality assurance (QA) and quality check (QC) criteria for human biomonitoring, generating standardized exposure metrics like biomarker concentrations. Strict biobanking protocols ensure secure storage, controlled access, and compliance with international standards for data integrity and reproducibility. Data is integrated with questionnaire responses via unique study IDs, enabling combined analysis for exposure-response assessments. Access in REDCap is restricted to authorized personnel, ensuring data security. Following cleaning and quality checks, combined biomonitoring and questionnaire data support robust analyses linking self-reported exposures to objective biomarkers.

##### 2.1.4.4 Data processing, analysis and standardization

In the HSSP, data is basically managed and processed via REDCap. This consists of standardized forms for data entry with validated fields to ensure data accuracy. REDCap can facilitate the automatization of data processing tasks by sending reminders or auto-calculating values. Additionally, REDCap facilitates data import and exports by supporting different data formats, and ensures data integrity with field validation. For data analysis, REDCap offers a set of basic analysis features. This mainly concerns summary statistics and visualizations, descriptive statistics parameters, stratified analysis based on variables, and direct data export to statistical software for advanced analysis. For data consistency and interoperability, REDCap provides standardized dictionaries that help defining data structure and terminologies, in addition to formatting rules for data normalization and automated data cleaning to minimize errors. The HSSP is a user friendly that is designed to be reachable both through Web and mobile versions, and it presenting an optimal environment to host projects and research studies aimed at assessing human exposure and potential health impacts. The HSSP includes several rubrics to provide the user with comprehensive information on the services provided as well as on the ongoing projects on the platform. This includes the objectives of the platform, scientific insights and publications on sentinel surveillance and its applicability in previous research, networks and projects using the platform and potential partners, in addition to sections that promote interaction with the user, namely, the Frequent Asked Questions (FAQ) and contact.

### 2.2 Workflow of HSSP as a tool for environmental and occupational health surveillance and research application

Ensuring a healthy environment and workplace is essential for public health and productivity. This framework applies sentinel surveillance to monitor environmental and occupational health risks, supporting early detection of hazards, tracking trends, and enabling timely interventions. By strategically selecting sentinel sites, collecting relevant health and exposure data, and integrating risk assessment with real-time reporting, this framework enhances our ability to identify emerging hazards, guide policy decisions, and implement targeted interventions. Its usability lies in its adaptability across different industries and environmental settings, making it a valuable tool for health professionals, policymakers, and occupational safety experts With this approach, we can strengthen preventive measures, reduce disease burden, and promote healthier workplaces and communities.

#### 2.2.1 Establishment of sentinel network

This consists of building a structured system that connects healthcare providers, epidemiologists, researchers, and public health officials to define the scope and objectives for the early detection, monitoring, and response to environmental and occupational health threats. The network pays also a crucial role in determining reporting strategies to inform policy decisions.

#### 2.2.2 Set-up of exposure assessment endpoints and objectives

This implies the determination of exposure biomarkers such as chemicals or environmental pollutants as well as the related health effect biomarkers. This will serve as a baseline to define the key aspects of the sentinel system and support the implementation as well as outcome investigation.

#### 2.2.3 Selection of sentinel sites

Concerns strategic locations, including group of individuals or populations, that can be used as early warning systems to detect and monitor health hazards and exposures. Sentinel sites can be health facilities tracking cases of diseases linked to environmental exposures or toxicities, workplaces that can monitor the exposure of workers to hazardous materials, occupational registries that can document occupational illness, public health centres or community-based monitoring sites that can assess the exposure of the general population to environmental pollutants and toxins. According to specific inclusion criteria, sentinel centers within the HSSP are designated: (i) being representative of high-risk or diverse exposure groups, (ii) being capable of using standardized biomonitoring and data protocols, (iii) complying with GDPR and ethical requirements, and (iv) being able to provide continuous longitudinal data. These criteria ensure that sentinel centers contribute high-quality, timely, and generalizable data to the surveillance network.

#### 2.2.4 Capacity building and training of health professionals

This training significantly enhances the effectiveness, accuracy, and impact of the sentinel system. Training of health professionals can have a great added value in environmental and occupational health risk assessment by increasing their ability to accurately detect, report, and analyse environmental and occupational hazards. Training health professionals also contributes to standardized data collection, improved trend analysis and effective risk communication, build capacity of the professionals which ultimately leads to a more efficient, data-driven, and sustainable surveillance system that informs policy decisions and protects public health.

#### 2.2.5 Data collection, analysis and reporting

This represents the crucial step in health risk assessment by generating important data that will guide thread detection and drive interventions. In this phase, the HSSP can significantly enhance the process of data entry and analysis, by providing a standardized, real-time platform for accurate reporting, ensuring timely interventions to protect public and workplace health.

### 2.3 HSSP applied to research projects for environmental and occupational health assessment

Currently, two projects are actively using the HSS platform. The first project is the Environmental Exposure Assessment Research Infrastructure (EIRENE RI), which is the first European infrastructure dedicated to the human exposome, and which aims to ensure the reproducibility of exposome research at EU level (21, 22). EIRENE-Flanders functions as the Belgian node within the EIRENE RI, drawing on existing expertise and analytical capabilities from initiatives like the Flemish Human Biomonitoring (FLEHS) program and the European Human Biomonitoring initiative HBM4EU (23–25). This infrastructure aims to assess the impact of chemical exposure on human health. In the line with these in a pilot study aims to connect occupational physicians and nurses to the EIRENE-Flanders network while testing the feasibility and usability of the HSSP. As part of this pilot project, the study will assess toluene exposure among Flemish workers during routine occupational health examinations. This will involve collecting exposure data from 400 employees with professional exposure to toluene, utilizing both biomonitoring techniques and a comprehensive questionnaire.

The platform is also used by the BIONET project which consists of an Euro-African Biomonitoring Network for the assessment of environmental exposure in population through universities and occupational health services (26). This project aims to reinforce capacities of African researchers and health professionals in occupational health and human biomonitoring as well as to implement a sentinel surveillance system in Africa, to assess the exposure of workers to hazardous chemicals and pollutants in workplaces. In this project, data is also being collected through the HSSP based on a survey questionnaire.

In these projects, the HSSP is allowing sentinel reporters to register and collect data. Consequently, this will lead to constituting networks of physicians, nurses and occupational health providers on a global level and will contribute to generating high quality data for exposome assessment in different regions of the world.

### 2.4 Users and beneficiaries of HSSP and public engagement

As a collaborative tool, the HSSP aims to foster collaboration among key stakeholders in occupational health and safety. The OHS professionals are central to the platform, contributing their expertise to collect and analyse biomonitoring and exposome data, thus contributing to public health. To enhance scientific understanding and to drive occupational health innovation, HSSP encourages collaboration with research institutions, including universities and specialized research bodies. As a result of anonymization and analysis, employees and workers benefit from strategies for mitigating health risks while maintaining privacy and ethical standards. By collaborating with the HSSP on research questions, commercial partners can contribute to industry-relevant advancements and support the research of the platform. Students can also conduct academic research in the HSSP, increasing their understanding of occupational health and reinforcing their skills. The stakeholders in this network are committed to improving workplace safety and advancing occupational health science. Importantly, HSSP also contributes to public health literacy and societal engagement. Sentinel surveillance frameworks increasingly incorporate community-based participants who provide biological or survey data and, in return, may receive aggregated feedback on exposure levels and health-related findings. This bidirectional flow of information enhances public understanding of environmental and occupational risks and can drive informed behavioural and policy responses. By enabling population-level awareness and promoting participatory risk governance, the HSSP has the potential to amplify the reach and impact of surveillance-derived health interventions, particularly in vulnerable or high-exposure populations.

## 3 Discussion

This study presents a novel contribution to the field by demonstrating how sentinel surveillance approach can be operationalized through a digital platform to address longstanding limitations in occupational and environmental health monitoring. Occupational and environmental health surveillance remains a cornerstone of public health practice. Also, rapid industrialization, technological advancement, and evolving work environments have introduced increasingly complex chemical, biological, and physical hazards into occupational settings integrating comprehensive, real-time, and multidimensional data on human exposures and health outcomes is essential to effective occupational and environmental health surveillance. However, existing sentinel systems often fall short in capturing the complex interplay between exposures and health outcomes in dynamic workplace environments, limiting their ability to identify cumulative risks or respond promptly to emerging threats. Several studies suggest that comprehensive surveillance systems, when equipped to handle diverse data sources, such as biomonitoring, surveys, and environmental data, provide rich insights into exposure-health linkages and enable more targeted preventive interventions (27–29).

In response to existing surveillance limitations, this study proposes the Human Sentinel Surveillance Platform (HSSP) as a robust, sentinel-based digital infrastructure designed to enhance occupational and environmental health monitoring. HSSP integrates multidisciplinary sentinel sites with real-time exposure monitoring, biomonitoring data, standardized metrics, and advanced analytics to support comprehensive risk assessment. By linking exposure data with health outcomes, the platform enables proactive hazard detection, evidence-based regulatory decision-making, and preventive health surveillance. Its scalable architecture offers a unified framework for high-resolution exposome data collection, management, and analysis, contributing to improved occupational health outcomes and strengthened surveillance methodologies. This discussion evaluates the potential impact and feasibility of the HSSP in improving health outcomes, informing regulatory frameworks, and advancing surveillance methodologies.

HSSP offers several critical advantages for monitoring occupational exposures and improving worker health outcomes. It aims to overcome limitations associated with traditional occupational health surveillance, which often focuses on single exposures or specific outcomes, leaving broader patterns and cumulative risks unexplored (30). By integrating multi-dimensional data streams, including exposure data, biomonitoring, lifestyle factors, and demographic data, the HSSP enhances the capacity to detect early health risk patterns and target interventions for high-risk groups. Additionally, integrating biomonitoring and real-time data collection allows for timely responses and informed decision-making, aligning with evidence indicating that early detection and continuous monitoring can substantially mitigate health risks in occupational settings (31, 32). Real-time data capture and analysis enable early identification of hazardous exposures, allowing for timely interventions to prevent health issues. This early detection is essential in mitigating occupational risks, as it provides the opportunity for targeted prevention before health problems escalate. Research has shown that early detection through surveillance systems significantly reduces the incidence of work-related diseases, highlighting the importance of proactive monitoring (27, 33, 34). Moreover, workplace surveillance practices have been shown to impact worker well-being, raising concerns about potential stress and privacy infringements (35). As Glavin et al. (35) emphasize, the balance between surveillance for safety and the potential psychological effects on workers is a critical issue that needs careful consideration.

Moreover, HSSP’s ability to collect exposure and health outcome data on a large scale supports comprehensive surveillance across multiple industries. This extensive dataset forms a solid foundation for robust epidemiological studies, enhancing our understanding of work-related health risks. Studies have demonstrated that large-scale data collection can reveal trends and patterns that inform effective interventions (24, 33). By analyzing comprehensive data on exposures and health outcomes, HSSP can identify high-risk worker populations and support tailored interventions. This data-driven approach provides policymakers with actionable insights, contributing to the development of evidence-based occupational health regulations and practices that enhance workplace safety (24, 25, 36).

In the developed HSSP, worker health surveillance is crucial for monitoring workplace injuries, illnesses, and exposures, aligning with NIOSH’s workplace surveillance system (28). This system not only identifies health issues but also addresses the environmental causes of work-related risks (National Institute for Occupational Safety and Health-NIOSH, 2019). Successful implementation relies on a multidisciplinary team as in the HSSP, where OHS professionals’ expertise ensures effective communication, exposure assessments, and data analysis48 HSSP also informs long-term safety policies by identifying health trends and guiding prevention strategies, ensuring proactive risk management and continuous safety improvements (28, 37, 38). Moreover, the HSSP is also highly adaptable for use across various countries and regions, including those outside the European Union. This flexibility is particularly beneficial for global occupational health surveillance, as it allows countries with diverse languages and regulatory contexts to participate in comprehensive exposome data collection.

### 3.1 Strengths and limitations

Establishing the HSSP across diverse settings may involve initial resource investment for training, data integration, and hardware requirements. Smaller organizations or those with limited occupational health infrastructure may face challenges in adopting the platform without additional support. Despite these limitations, HSSP represents a significant advancement in occupational health monitoring, filling critical gaps in exposome data collection and analysis and promoting healthier work environments through informed interventions and policy.

The HSSP promptly informs employees of their results, explaining their significance and recommended next steps. Significant findings are communicated to employers and, when necessary, to regulatory bodies, in full compliance with privacy regulations. However, a current limitation of the HSSP is the lack of an automated notification feature; this functionality, crucial for enhancing timely communication, is planned for future implementation.

### 3.2 HSSP contributions to public health policy and practices

Application of sentinel surveillance in health practice is driven by the need for a robust network of health care professionals dedicated to monitoring occupational and environmental exposure and improving workplace health. This approach also enables alignment with broader European initiatives, such as *Human Biomonitoring for Europe* (HBM4EU) and the *Partnership for the Assessment of Risks from Chemicals* (PARC). These initiatives promote standardized data collection and sharing, strengthening the capacity for coordinated occupational health surveillance across Europe (24, 25, 39).

The HSSP consolidates occupational exposure and health data to improve risk assessment, support regulatory decisions, and promote proactive worker health protection. With capacity-building initiatives and standardized data protocols, it helps stakeholders identify trends, respond to emerging hazards, and implement targeted interventions for enhanced workplace safety. Its adaptable framework supports diverse industries, fostering a prevention culture across various settings. By integrating advanced technologies, HSSP enhances early detection, informs evidence-based interventions, and advances exposome research. Expanding its scope to include the general population in exposure assessments could reduce chronic and acute diseases linked to environmental exposures, making HSSP a key tool for both occupational health and public health resilience, improving safety and quality of life globally.

## 4 Conclusion

The HSSP offers a scientifically grounded and adaptive framework for advancing occupational and environmental health surveillance, fostering a more proactive and systematic method for managing health risks. By leveraging integrated data sources and advanced technologies, it not only improves hazard detection but also enables more precise and timely interventions. The collaboration of diverse stakeholders and multidisciplinary institutions within the HSSP framework ensures that health surveillance evolves from reactive to preventive, ultimately enhancing both individual and population-level health outcomes. Its potential to promote data-driven policy and health strategies positions the HSSP as a crucial tool for future public health resilience and sustainable environment. The HSSP addresses critical limitations in occupational and environmental health surveillance by providing a validated, real-time platform for exposure assessment. Its demonstrated ability to reduce data latency and identify high-risk populations strengthens its potential as an evidence-based tool for regulatory and policy application. Future research should focus on expanding biomonitoring panels to include emerging contaminants aligned with international exposure assessment priorities (e.g., PFAS) and on implementing automated hazard alert systems to improve the platform’s responsiveness and preventive impact.

The HSSP is designed as an open, collaborative platform that welcomes engagement from researchers, public health authorities, and industry partners, providing a foundation for future projects and joint initiatives in exposure science and health surveillance.

## Data Availability

Data can be made available upon request to the corresponding author.
