# Correction: A human sentinel surveillance framework for comprehensive exposure assessment in occupational and environmental health

**DOI:** 10.3389/fpubh.2025.1735989

**Published:** 2025-11-18

**Authors:** Emine Aktas, Kaoutar Chbihi, Hilde De Raeve, Janne Goossens, Lode Godderis

**Affiliations:** 1 Independent Researcher, Istanbul, Türkiye; 2 Environment and Health Unit, Department of Public Health and Primary Care, Faculty of Medicine Katholieke Universiteit Leuven, Leuven, Belgium; 3 Human Epidemiology and Environmental Health Team, Faculty of Sciences, Moulay Ismail University, Meknes, Morocco; 4 IDEWE, External Service for Prevention and Protection at Work, Heverlee, Belgium

**Keywords:** environmental exposure, occupational exposure, exposure assessment, human biomonitoring, exposome, sentinel surveillance, risk assessment, data analysis

Author Emine Aktas was erroneously spelled as Emine Aktaş.

Affiliation “Independent Researcher, Istanbul, Türkiye” was omitted for author Emine Aktas. This affiliation has now been added for author Emine Aktas.

The funder European Union's Horizon Europe research and innovation programme under Grant Agreement no 101057014 (KU Leuven internal project code: 3M220270) was erroneously omitted. The correct **Funding** statement appears below:

The author(s) declare that financial support was received for the research and/or publication of this article. The HSS platform was developed within the framework of the EIRENE-RI project as EIRENE-Flanders—the Flemish hub of the International Exposome Research Infrastructure EIRENE—funded by the Research Foundation Flanders (FWO) International Research Infrastructure (IRI) project (grant no. I001923N), and the study is also part of the Partnership for the Assessment of Risk from Chemicals (PARC), funding by the European Union's Horizon Europe research and innovation programme under Grant Agreement no 101057014 (KU Leuven internal project code: 3M220270).

The original version of this article has been updated.

